# Understanding the needs and perspectives of young adults with recent suicidal ideation: insights for suicide prevention

**DOI:** 10.3389/frcha.2024.1376872

**Published:** 2024-06-12

**Authors:** Milou Looijmans, Elke Elzinga, Arne Popma, Diana van Bergen, Renske Gilissen, Saskia Mérelle

**Affiliations:** ^1^ Research Department, 113 Suicide Prevention, Amsterdam, Netherlands; ^2^ Department of Psychiatry, Amsterdam UMC, Amsterdam, Netherlands; ^3^ Faculty of Pedagogical and Educational Sciences, University of Groningen, Groningen, Netherlands

**Keywords:** suicide prevention, young adults, needs, perspectives, suicidal ideation (SI)

## Abstract

**Introduction:**

Suicide rates among adolescents and young people are increasing, especially in Western countries. Suicidal ideation often precedes suicide attempts and suicide. Yet, research involving individuals with lived experience in suicide prevention, especially among young adults, remains scarce. Understanding their needs is crucial for effective interventions. This qualitative study aims to explore the needs and perspectives of young adults with lived experience to provide tailored recommendations for suicide prevention.

**Methods:**

Semi-structured interviews were carried out with 19 young adults who had experienced suicidal ideation within the past two years. Open-ended questions addressed the needs for help and support regarding suicide prevention. Data was thematically analyzed and, through an iterative process involving discussion among all authors, categorized into six themes.

**Results:**

The results indicated needs around more openness and understanding of suicide among the general public, advocating mental health education starting from a young age, reducing barriers in mental health care such as long waiting lists and enhancing informal support systems by facilitating online and offline peer connections. Participants also highlighted contemporary concerns such as social welfare, academic pressure, and social media as significant needs in the current time.

**Conclusion:**

This study highlights the necessity for comprehensive suicide prevention approaches catering to the diverse needs of young adults with recent suicidal ideation. It highlights the urgency of societal awareness, early mental health education, and improved access to services. Informal support networks and addressing societal stressors are also deemed crucial. Structural changes are urged to create supportive environments.

## 1 Introduction

Suicidal ideation and suicidal behaviors are important public health concerns (1). Rates of suicide among adolescents and young individuals are on the rise in numerous Western countries (2–7). However, fatal suicides represent only a fraction of the overall burden of suicidal behavior, with an average of twenty suicide attempts for each fatal suicide (8) and a larger number of individuals experiencing suicide ideation (9). Suicide attempts and suicidal ideation not only contribute to the total burden but also serve as important risk factors for suicide (10).

Among all age groups, young adults exhibit the highest incidence of suicide attempts (10). Alarmingly, in the Netherlands, where this study was conducted, there has been a rise in suicides among young people, particularly those aged 20–30, whereas other age groups remained stable or even exhibited a slight decrease (11). Also non-fatal forms of suicidal behaviour increased within this population in the past years. Presentations of self-harm behaviour at Emergency Departments (ED) of hospitals, which functions as a proxy for suicide attempts, increased in recent years (12). Furthermore, in December 2023, 15% of young people aged 18–25 in the Netherlands reported seriously considering suicide in the past three months, nearly double the rate compared to two years prior (13) and also almost doubling the rate of adults over the age of 25 years (14).

Since 2014 the Netherlands has adopted a National Agenda for suicide prevention, an instrument to achieve strategic goals in terms of suicide prevention (15). Additionally, a national helpline and extensive research program are in place (16). The ratio between the numbers of suicide within and outside specialized mental health care has remained consistent over the past decade with approximately 40% of suicides occurring in individuals receiving specialized mental health care at time of their death and 60% who were not (17). Compared to a neighboring region (Flanders, Belgium), the Netherlands shows higher rates of seeking professional help for psychological problems. Moreover, the Netherlands demonstrates a relatively accepting and less stigmatizing attitude towards suicide (18, 19). Despite generally positive attitudes toward mental health care, some taboos persists. Ongoing efforts of the suicide prevention strategy are, among others, to further destigmatize mental health issues and promote suicide prevention in the Netherlands (20).

There is a growing recognition on the importance of involving individuals with lived experience in the development of suicide prevention interventions. This is crucial for ensuring that prevention efforts align with their needs, likely enhancing its effectiveness. Young people up to 25 years with suicidal behaviour, for example, value informal support (such as friends and other peers) (21) and find it important to be able to talk to peers with lived experience in professional counseling (22). However, current research is limited in reporting engagement of individuals with lived experience of suicidal ideation/behavior in suicide prevention efforts (23). Recently, Krysinka et al. (2023) published recommendations on the active involvement of individuals with lived experience of suicide in suicide research, advocating for co-production (24). Despite the importance of involving young adults with lived experience in suicide prevention being increasingly recognized, there is still a significant scientific gap, with very few studies worldwide in this area (23).

Because of the increase in suicides among young adults aged 20 to 30 in recent years (2–7), it is important to gain insights into their experiences and needs in terms of suicide prevention. With increased understanding of the experiences and needs of young adults experiencing suicidal ideation, intervention and prevention efforts can be better aligned. Therefore, this study aims to answer the question; “What are the needs and perspectives in terms of help and support for young adults with recent suicidal ideation to prevent suicidal behavior?”

## 2 Methods

### 2.1 Ethics

According to the Dutch Medical Research Involving Human Subjects Act this study was exempted from ethical review as decided by the Medical Ethical Committee of Amsterdam UMC (registration number 2022.0771). All participants provided written informed consent.

### 2.2 Study design and procedure

This study was part of a larger research project that also assessed background factors and environmental stressors associated with recent (up to two years ago) suicidal ideation, with the aim of making recommendations to address the increasing trend of suicides among young adults in the Netherlands (25). Semi-structured interviews were conducted with 19 young adults aged 20–30 years who had experienced suicidal ideation within the past two years. Data collection took place from April to May 2023.

We used a convenience sample to select young people with lived experience in suicidal ideation. Initially, we posted a call for participation on the LinkedIn page of 113 Suicide Prevention to invite young adults with lived experience to take part in the study. Individuals could express their interest by completing a form. Within a short timeframe, this generated more responses than anticipated (*n* = 59); consequently, we closed the call shortly afterward to evaluate the suitability of candidates for participating in the interviews. We sent them emails in the order of their registration to assess whether they met our inclusion criteria (aged between 20 and 30 years, residing in the Netherlands, fluent in Dutch, and not experiencing acute suicidal thoughts). Due to a significantly higher number of female respondents, we selected the female respondents in the order of their registration who met inclusion criteria (*n* = 13) and all male respondents who met the inclusion criteria (*n* = 7). Additionally, the participants were provided with the information letter, informed consent form, and several interview appointment options. Participants who had additional inquiries or for whom it was uncertain whether they were experiencing acute suicidal thoughts were contacted by phone prior to scheduling an appointment. Throughout this process, some respondents (*n* = 4, 1 male and 3 female) dropped out either due to finding it too burdensome or by ceasing to respond altogether. We proactively sought male participants to ensure a balanced gender representation and reached out to organizations serving young adults with lived experience as well as leveraged the personal networks of the researchers, ensuring an indirect approach to avoid any direct relationships. This effort yielded 3 extra male participants. Initially, our aim was to include approximately 12–16 participants, a number typically deemed sufficient for data saturation to occur in qualitative studies (26–28). We ultimately included 19 participants; 9 males and 10 females.

Participants were asked to return a signed informed consent form and indicate their preference for either an online or offline interview. As all participants expressed a preference for online interviews, all interviews were conducted and recorded via Microsoft Teams. Immediately before the interviews, participants were asked about their current experience of suicidal thoughts. Participants who reported acute suicidal ideation at that time would have been excluded from the study, yet this scenario did not arise.

The interviews were conducted in Dutch and lasted an average of 37 min, ranging from 20 to 53 min. All interviews were conducted by one of the two researchers from the project team (ML or EE; respectively MSc clinical psychologist, and PhD, both female research employees 113 Suicide prevention with approximately 4–5 year experience in qualitative research), with assistance from a research assistant (MJ or MG; respectively female and male, both BSc clinical psychology), who observed, made field notes and sometimes asked follow up questions. Before commencing the interviews, the researchers explained their background and the reasons for conducting this study. Participants received a 20 Euro voucher as compensation for their participation.

### 2.3 Measures

As our goal was to delve into participants’ needs based on their personal narratives and experiences, we conducted qualitative semi-structured interviews. We used an open interview topic guide, starting with a broad open-ended question addressing the needs for help and support in suicide prevention. This was followed by numerous probing questions to uncover what had helped participants during difficult periods, what they felt was absent, how they perceived others could provide assistance, and in what manner. All interviews were recorded and transcribed verbatim. The interview topic guide is available in Supplementary Material S1.

### 2.4 Analyses

The data were independently coded by two members of the coding team to enhance the reliability of the coding process. The coding team comprised two researchers (ML and EE) and two assistants (MJ and MG). The researchers initiated the coding process by performing inductive coding on the first four interviews, resulting in the creation of a code list. Subsequently, all four members of the coding team proposed modifications and additions to this code list through discussions conducted as the coding process progressed. Thematic analysis of the data was conducted using the constant comparison method using Atlas.ti (29, 30).

The coded fragments were extracted per topic, and the results were summarized per theme. Subsequently, a matrix was constructed, with cases represented in the rows and various output variables, such as different wishes and needs, represented in the columns. The matrix was filled with summaries of the coded excerpts per case (row). Through an iterative process and discussion among all members of the coding team, the results were grouped into six categories. For the purpose of this paper, the categories and quotes were translated into English.

This study was conducted in line with the 32-items Consolidated criteria for Reporting Qualitative research (COREQ) (31) (Supplementary Material S2).

## 3 Results

### 3.1 Population characteristics

Nineteen young adults with recent (within the past two years) suicidal ideation were interviewed (see Table 1). Participants were between 20 and 28 years old with a mean of 24 years. Of the 19 participants, nine were male and ten were female. About one in four identified themselves as LGBTIQA + (*n* = 6). Their highest level of education ranged from “vocational school” (education focusing on providing practical skills and training for specific careers or trades) (*n* = 7) to “universities of applied sciences” (higher education focusing on practical education and preparing students for specific professions and industries, often offering bachelor's degrees) (*n* = 4) to “academic education” (education focusing on theoretical knowledge, research, and academic formation, offering programs at various levels, including bachelor's, master's, and doctoral levels) (*n* = 8). The onset of suicidal ideation ranged from childhood to 24 years. In most cases, this was preceded by several years of depressive episodes or other psychological problems.

**Table 1:** Population characteristics.

	Gender	Age range	LGBTIQA+	Level of education	Source of income
1	M	25–30	No	Vocational school	Unemployement benefit
2	F	25–30	Yes (B)	Vocational school	Work
3	F	25–30	No	Academic education	Student loan/side job/parents
4	F	25–30	No	Academic education	Side job
5	M	25–30	No	Academic education	Work
6	F	25–30	No	Academic education	Work
7	F	25–30	No	Universities of applied sciences	Parents
8	M	20–24	Yes (T)	Vocational school	Work
9	F	25–30	Yes (L)	Universities of applied sciences	Work
10	F	25–30	Yes (B)	Universities of applied sciences	Student loan
11	M	20–24	No	Vocational school	Student loan/side job
12	F	20–24	No	Vocational school	Work
13	M	20–24	Yes (B)	Vocational school	Work
14	F	20–24	No	Academic education	Student loan/side job
15	F	20–24	No	Universities of applied sciences	Work
16	M	25–30	Yes (G)	Academic education	Work
17	M	20–24	No	Vocational school	Work
18	M	25–30	No	Academic education	Side job
19	M	20–24	No	Academic education	Student loan/side job

### 3.2 Psychological problems

All 19 participants received professional mental help for psychological symptoms at some point in their lives. This was not always related to suicidal ideation and sometimes took place much earlier in life. Diagnoses included (a combination of) Post-Traumatic Stress Disorder (PTSD; *n* = 9), depression (*n* = 7), Autism Spectrum Disorder (ASD; *n* = 4), Attention Deficit (Hyperactivity) Disorder [AD(H)D; *n* = 4], eating disorder (*n* = 4), anxiety disorder (*n* = 3), personality disorder (*n* = 2), schizophrenia (*n* = 1), emotion regulation disorder (*n* = 1), and Tourette syndrome (*n* = 1). Diagnoses were evenly distributed between male and female participants, except that women were more frequently diagnosed with eating disorders and anxiety disorders than men. Although mental health issues were present, the participants mostly did not identify them as the direct cause of their suicidal ideation.

### 3.3 Needs and perspectives

Upon analysis, it became evident that our data extends beyond discussion of personal needs to more general ideas. In our results we include both perspectives, and we make every effort to describe them as conveyed and intended by the young adults in this study. Furthermore, the findings revealed instances where discussions on suicide needs or prevention seamlessly transitioned into broader conversations about mental health, sometimes intertwining the two. Therefore, in presenting the results, we strive to accurately describe the intended focus whenever possible. The needs and perspectives of young adults who experienced recent suicidal ideation could be grouped into the categories and subcategories shown in Table 2.

**Table 2:** Categories and subcategories of needs and perspectives of young adults with recent suicidal ideation.

Categories and subcategories
1. More openness and knowledge about suicide among the general public
2. Mental health education
3. Facilitating seeking help for mental health care
4. Reducing barriers in mental health care
- *Discussing suicidal thoughts within therapy*
- *Accessible and available mental health care*
- *More peer specialists*
5. Improving informal support
- *Support system in school and at the workplace*
- *Facilitating (safe) places to connect*
- *Having a purpose and self-help*
6. Addressing societal stressors
- *Improving social welfare*
- *Reducing academic pressure*
- *Social media*

#### 3.3.1 More openness and knowledge about suicide among the general public

Many participants emphasized the importance of fostering greater openness regarding suicidal ideation. One of the key needs highlighted by participants was the need to break taboos surrounding these topics, with the goal of promoting and facilitating discussions about suicide within communities.

*“For me, there was a huge taboo about talking about it [suicidal ideation]. I believed I was alone in my thoughts, feelings, and experiences, and that I shouldn't share them with anyone. I wasn't aware that many others were also struggling with similar issues. If I had known, perhaps I would have reached out to someone. However, since I never saw these discussions happening, I remained silent. If conversations about suicidal ideation were more common, perhaps I would have sought help sooner.”*

Several young adults shared similar experiences, recounting instances where they disclosed their suicidal thoughts, eliciting shock or caused others to distance themselves. As a result, individuals felt apprehensive to discuss their suicidal thoughts, fearing these overbearing reactions or judgment from others. To address these challenges, participants expressed a need for greater knowledge and information, particularly within the social circles of individuals experiencing suicidal ideation, on how to talk about suicide. Participants expressed a need for individuals being present, listening, to hear them without judgment and offering support without feeling compelled to take specific actions.

Participants emphasized that everyone can play a role in suicide prevention, not solely healthcare professionals. According to young adults, suicide prevention campaigns should not only focus on individuals experiencing suicidal thoughts but also engage their surrounding communities. This approach seeks to foster open dialogues about suicidal feelings among all members of society.

#### 3.3.2 Mental health education in schools

Almost all participants stressed the importance of integrating mental health education into the curriculum from an early age, such as in elementary or secondary school. They perceived a current lack of discussion about mental health in schools, despite it being a setting where many individuals first encounter challenges. Participants stated that because mental health is seldom discussed in schools, it's challenging to take the first step and talk about it when struggling with an issue, despite that many others face similar challenges. Participants expressed not only a need for greater understanding of suicide through education in schools, but also a broader need for increased awareness and knowledge of mental health overall, both for themselves and for others. The participants unanimously agreed that education plays a crucial role in this matter and that suicide prevention and especially mental health in general should be an important topic in schools from an early age.

*“…there should be more attention on this topic in schools because, of course, there are many lessons on things like sex education, but I've never had a lesson on mental health issues or how to deal with them. Really none.”*

*“I think we also need to look at education because it really shouldn't be just general education anymore. It's not just about math, languages, and biology. I believe we should also focus much more on those societal issues [suicide prevention and mental health in general] and that there should be much more attention given to them.”*

#### 3.3.3 Facilitating seeking help for mental health care

Many young adults mentioned that they struggled with seeking help indicating an unmet need. This was sometimes because they did not realize they needed help, and other times because they did not know where to turn. Participants reported having no oversight of what kind of help is available, or what a help trajectory looks like. Some mentioned that they were dependent on others to direct them towards help-services:

*“At one point, my boss said, “You'll be off work for at least a month. You're going to call your parents now because you're not doing well, and if you don't call them, I will.” To me, that had a significant impact, because I was like, okay, they even notice this at work.”*

Furthermore, there were certain beliefs among young adults that hindered them from seeking help, such as the fear of being immediately admitted to a clinic when they would mention having suicidal thoughts or behaviors. There expressed a need for a more accurate understanding of the available support services.

Regarding mental health care, young adults mentioned the importance of having clear information about when and where to seek help, as well as the need to facilitate the process of seeking professional assistance. Some suggested providing concrete examples of situations warranting help and offering an overview of waiting times for services, as these measures could help individuals take the initial step towards seeking help.

*“That there is just a clear overview of existing support like: we have this kind of support in educational settings. There you have to wait for two years. We also have professional mental health care. Therefor you have to go to the general practitioner. There you wait for so long, and we have this, this and this. There you often wait a little less long, but you pay more. I mean, just something, an overview, like that.”*

#### 3.3.4 Reducing barriers in mental health care

##### 3.3.4.1 Discussing suicidal thoughts within therapy

Almost all participants mentioned that the therapy they had received had been a crucial factor on their road to recovery. Most participants reported that a connection with the therapist was among the most important factors for therapy to be helpful. One of the main needs they reported within their treatment, was the ability to discuss suicidal thoughts. Many participants reported experiences with health care professionals where they felt no space to open up about their suicidal thoughts. Some participants reported being open, but felt like the health care professional did not genuinely listen or make them feel heard. They reported experiences like health care professionals being concerned about practical matters or safety measures, but avoided the “real” conversation, while especially this—having an open conversation about their suicidal thoughts—was a significant need for the participants. Some participants reported negative experiences with health care professionals like not feeling taken seriously when discussing their suicidal thoughts, not being treated with respect, feeling judged, or not getting sufficient time and attention.

##### 3.3.4.2 Accessible and available mental health care

Other more general needs mentioned very often included addressing the issue of waiting lists and providing transitional care in between treatments or health care facilities. Furthermore, there was the need that had to do with the accessibility of healthcare professionals. While participants understood that healthcare professionals were not available 24/7, their suicidal thoughts did not always occur during regular office hours. The option to contact healthcare professionals outside of working hours, with clear arrangements, was therefore very helpful.

##### 3.3.4.3 More peer specialists

Many participants expressed a need for more peer specialists in mental health services because they understand them, and they found it easier to talk to people who have experienced the same things.

*“I found it especially important to have someone who knew what I was talking about, like a kind of peer specialist. I found that very comforting because, you see, a regular psychologist can make an assessment of how you're feeling, but they don't really know how you feel. A peer specialist actually knows exactly how you feel because they've been through it themselves, and that's so comforting, just to talk to someone who really understands you. I found that very comforting…”*

#### 3.3.5 Improving informal support

##### 3.3.5.1 Support system in school and at the workplace

Regarding accessible support in education, it was mentioned that while academic advisors are familiar to most participants, young adults felt that they were not always sufficiently equipped to handle complex mental problems. Although some educational institutions have student psychologists (often, but not always, present in both secondary and higher education), who seem well-suited, participants mentioned that sometimes there are long waiting lists or they still feel a large barrier to approach them. As a result, young adults expressed a general need for individuals in educational institutions who are easy-accessible and trustworthy, “like mentors in high school,” to turn to when facing problems. A mentor in Dutch secondary school is typically a teacher who provides guidance, support, and advice with academic, personal, and social matters to a group of students.

Furthermore, workplaces and educational institutions could play a more significant role in disseminating information about where to turn when seeking (easily accessible) help, such as certain online platforms or support groups. There was the need for every employer to have a designated person to go to and a clear protocol for employees dealing with mental issues in the workplace.

##### 3.3.5.2 Facilitating (safe) places to connect

Many participants with recent suicidal ideation expressed a need for (more) contact with peers. Participants appreciate that peers have similar experiences, which provide a sense of recognition and make conversations easier.

Some young adults explicitly mentioned the need for an informal place (online or offline) where safe and hopeful messages or stories can be shared, where they can exchange and connect with peers.

Participants mentioned several times that a physical place, where you can step away from your own environment and talk to others, such as peers and possibly a professional, would be helpful. When the interviewers probed further, asking if, for instance, they would consider utilizing a mental health drop-in center (a welcoming space designed to provide mental health support and resources in an informal and accessible setting), they responded that although they found these good initiatives, they would not use them themselves. They perceived a significant barrier and had the idea that these drop-in centers were primarily intended for people with severe mental problems, which they did not identify with. Online forums were mentioned several times as informal places helping young adults with mental issues express and ventilate their feelings. Altogether, participants seemed enthusiastic about initiatives that promote discussions about suicidal thoughts or mental issues without the need to visit a professional doctor or psychologist.

##### 3.3.5.3 Purposeful activities and self-help

A few young adults mentioned that engaging in meaningful or purposeful activities, such as volunteer work, really helps them to continue feeling well. They also noted that having a specific goal or mission, where they find meaning or purpose, is helpful. Also, some participants wanted to use their own experiences to help others or to advocate for those who are no longer here. For some participants, especially understanding oneself and recognizing their own, personal triggers was very helpful. For some participants, especially understanding oneself and recognizing their own, personal triggers was very helpful.

#### 3.3.6 Addressing societal stressors

##### 3.3.6.1 Improving social welfare

Some mentioned that it would be helpful if certain (major) societal problems, such as the housing crisis or inflation, were resolved or improved. Additionally, increased social welfare was mentioned as a potential preventive measure. It would, for example, be better if some students, due to mental health issues, would not have to choose between receiving benefits or pursuing their education. According to participants, it would be better if everyone could participate in society at their own pace and ability, and no one was excluded if they could not keep up.

##### 3.3.6.2 Reducing academic pressure

Many participants mentioned that, concerning education, it would have helped them personally if there had been more tailored support and greater emphasis on individual solutions when facing challenges in their studies due to mental health issues. Young adults expressed a desire to receive more time, assistance, or understanding, for instance, rather than having to abruptly discontinue their studies.

*“I think what also played a huge role for me is the pressure from my studies. Especially when I was studying [Field of study], we had very long days, we spent a lot of time in the [detailed information removed]. […] That was just very tough for me. And then I did ask for help several times, for example from study advisors, but actually there was quickly nothing possible and everything is mandatory, just make sure you do it, and if you are absent more than once then you can come back next year. And that gave me a lot of pressure because I just kept thinking: okay, I have to endure this.”*

##### 3.3.6.3 Social media

Some participants mentioned that they had needed the ability to share on social media about their mental health problems in a safe manner rather than in a destructive way. It was mentioned several times that ideally, there should be more education about the dangers of social media. This applied to both general aspects of social media that increase stress for young adults, such as social comparison, as well as explicit suicidal content. For instance, there should be warnings on social media that advise caution when viewing certain content if you are not feeling well. It was also suggested that teaching young adults how to reconnect with each other without relying on social media could be helpful. According to participants, it would help if people on social media were more authentic and showed variations in how life is supposed to be or the vulnerable aspects of life. In addition, positive videos were mentioned as a counterbalance to suicidal content:

*“Now, there are a lot of videos going around like, “I'm lonely, so I'm jumping off a bridge.” But there aren't counter-videos that say, “I wanted to do this, but I got help. So, things are better now. I think there's a big gap there.”*

On the other hand, many participants mentioned that they also learn a lot about mental health through social media and that it therefore can be used as a channel for education and information. One participant began to recognize signs of mental health problems through following a well-known social media influencer, which ultimately led to starting the journey to seek help.

#### 3.3.7 Discussion

This interview study was conducted to answer the question: “What are the needs and perspectives in terms of help and support for young adults with recent suicidal ideation to prevent suicidal behavior?” The results showed that the young adults in this study have general needs in terms of breaking taboos and facilitating discussions about suicidal behavior and mental health, as well as specific requirements regarding education and healthcare. The needs of young adults with recent suicidal ideation do not appear to differ much from the needs of adolescents with suicidal behavior (32). Also older adults (75+) with prolonged death wishes) seem to share the need for meaningful conversations with caregivers and recognition and understanding also play a significant role (33). A plausible difference between the needs of younger generations and older generations could lie in the realm of social media and social welfare related needs, although more research is needed to compare needs across generations.

##### 3.3.7.1 More openness and knowledge about suicide among the general public

The most important needs of the participants in this study were related to more openness around suicide and mental health problems in general, and thereby making it easier to talk about suicidal ideation. Corresponding with other literature about the needs of young adults regarding mental health in general (34), young adults in this study expressed a need for social connection and being heard when talking about their suicidal ideation. The wish to inform and educate individuals within the environment of young adults about initiating conversations about suicide and remaining aligns with one of the goals of the Dutch national suicide prevention agenda: “encouraging and learning to talk about suicide” (15). A freely accessible online suicide prevention training has been launched, aimed at instructing individuals on how to initiate discussions about suicidal thoughts and potentially offer assistance (35).

##### 3.3.7.2 Mental health education in schools

There was a strong wish for education on suicide prevention and mental health from a young age. In the Netherlands, there are several projects focusing on providing mental health education for students (36–38). However, whether or not students receive this education often depends on the individual school's interest in participating in such projects. Educational institutions often struggle to identify effective interventions and strategies aimed at enhancing the mental well-being of students, and face challenges in implementing these programs. Hence, there is a pressing need for more studies on the effectiveness of interventions and programs, as well as into implementation strategies (39–43). Integrating suicide prevention and mental health education into the curriculum would be beneficial to ensure education for all individuals on these matters. Globally, there is an increasing focus on peer education, where peers educate their fellow peers regarding various health-related topics. This approach receives significant support, likely because young people tend to favor seeking help for mental health issues from their peers rather than from adults or professionals (44), as also evidenced by the results of this study.

##### 3.3.7.3 Facilitating seeking help for mental health care

There appeared to be a need for greater clarity regarding the pathway to seeking help and for a more concrete and accurate understanding of existing mental healthcare and treatment options. Although almost all participants had received help for mental health issues at some point in their lives, they found it difficult to access help specifically for their suicidal thoughts. While there is a lot of information available on the internet about accessing and navigating mental health care, participants suggested a preference for actively providing this information at specific moments in life, such as during their school or study period, and specifically addressing the pathway for seeking help when experiencing suicidal ideation.

Interestingly, the primary barriers for young people to seek mental health care in general are not only related to the pathway to help, but according to Gulliver et al. rather to the perceived amount of stigma and embarrassment, the difficulty of recognizing symptoms, and the preference for self-reliance instead of professional help (45). The young adults from the current study also reported these barriers. Providing education, both to young adults and their communities, as well as to (mental) health care providers generally, can generally help fight stigma, embarrassment and challenges associated with recognizing mental illness (46). Additionally, providing self-help therapy programs to cope with suicidal thoughts can help meeting the needs of young adults, while taking into account their desire for self-reliance (47).

##### 3.3.7.4 Reducing barriers in mental health care

A large part of the needs had to do with the connection with young adults’ mental health care provider and the need to be heard and discuss suicidal feelings in a space where they could share their story without an immediate focus on safety measures and other interventions. A review by Lynch et al. has already demonstrated that aspects of the relationship between a healthcare provider and young individuals (aged 10–24 years), such as trust and confidentiality, as well as qualities of the healthcare provider such as understanding, caring, openness, nonjudgmental, and a shared decision-making process are crucial for engaging and maintaining young people in mental health care (48). For young adults with suicidal ideation in particular, the relationship and the space the therapist creates to listen openly and without immediate action to the suicidal narrative may be crucial. For professionals, however, it is often difficult to engage in “therapeutic risk-taking,” where coercive measures to ensure safety are avoided and instead, more space is allowed for patient autonomy. It is important for therapists to maintain this balance to optimize the therapeutic relationship and facilitate recovery (49). Also often mentioned was the need to talk with professionals with lived experiences besides regular therapists. This need also arose in a number of studies of adolescents with suicidal behavior (32); however, while people with lived suicidal experience are increasingly being employed in health care, there is not yet much known in the scientific literature about how to do this in the most safe and effective way. Employing peer specialists offers many advantages, such as recognition and someone who truly understands the suicidal ideation but, at the same time, it is important to keep in mind the emotional burden that comes with working with clients who are suicidal for the peer specialists (50). Nevertheless, incorporating peer workers within community and clinical mental health services has been shown to improve service delivery outcomes for users (51).

##### 3.3.7.5 Improving informal support

There were notable needs for more personalized support during education and easily accessible contacts within school- as well as in work environments. The World Health Organization (WHO) has released guidelines on mental health at work (52). These provide recommendations including organizational and individual interventions, training managers and workers, and on how to return to work or gaining employment. These guidelines help organizations to provide an inclusive workplace and enable people living with mental health conditions to participate and thrive at work.

However, it is of course also crucial not to overlook young adults who are not in a school or work environment (53). Especially since many study participants reported challenges in participating in either their educational pursuits or employment while experiencing suicidal ideation. Furthermore, a frequently mentioned need concerning informal support was the desire for (more) contact with peers, both offline and online. This aligns with studies indicating that for young adults in general, as well as for those with lived experience of suicidal ideation or self-harm, friends (and family) are crucial sources of support (44, 54). Consequently, peers can play a significant role in determining whether or not to seek professional help (44, 54).

Peers as gatekeepers have potential, but recent reviews indicate insufficient research on their effectiveness (55). Participants in this study also highlighted the importance of online forums for sharing experiences with suicidal thoughts. Online forums provide young individuals with a platform to access emotional or informational support, with the option of anonymity if desired. However, challenges persist in managing service users’ expectations, ensuring reliability of information, maintaining consistent support, and preventing the normalization of unhelpful messages (56). Nevertheless, these forums offer valuable support, particularly for those who do not seek professional help. In the Netherlands, initiatives like “Letztalk café” provide offline spaces to discuss suicidality, but scientific evidence supporting these initiatives is lacking (57).

##### 3.3.7.6 Addressing societal stressors

Another substantial part of the identified needs were about more structural changes within society like improved social welfare and less academic pressure. Social determinants, structural inequalities, and social context have a well-known substantial impact on mental health (58). Regarding societal stressors the results showed several needs concentrated around social media use. Previous research into the social media habits of young individuals who have died by suicide brought to light frequent discussions among the bereaved regarding the adverse impacts of social media such as dependency, triggers, imitation, challenges, cybervictimization, and psychological entrapment (59). Despite the strength of the evidence not being unequivocal (60), various reviews suggest that social media use among young people is associated with an increase in mental distress, self-harming behaviors, and suicidality (61, 62). Solutions for the minimization of social media harm are not simple but appear to involve, at the very least, a shared responsibility between governments and social media companies (63). A prime example of an intervention for fostering safe online conversations is Chatsafe, which provides guidance to young people on discussing self-harm and suicide in a secure manner across social media and other digital platforms (64).

##### 3.3.7.7 Strengths and limitations

The major strength of this study is that it provides recommendations for suicide prevention that are derived directly from the statements of young adults with who recently experienced suicidal ideation. The qualitative interview data provided rich insight into the experiences and needs of this vulnerable group. However, there were also limitations. First, this study consisted of semi-structured interviews with 19 participants. It's important to acknowledge that while 19 participants may provide valuable insights, there are inherent constraints. The relatively small sample size may limit the diversity and representativeness of the participants, potentially impacting the generalizability of our findings. Taking into account that saturation was reached after 19 interviews, no new themes or insights emerged from additional interviews, indicating that the sample size was adequate for the study's objectives. However, while saturation provides confidence in the depth of understanding within the sample, it does not necessarily address concerns about the sample's representativeness or generalizability to broader populations and therefore a larger sample would have been desirable. In addition, since most (16 out of 19) participants responded through the LinkedIn of 113 Suicide Prevention there is a possible bias of participants who were already interested in 113 and active on this particular platform. This may limit the generalizability of the results because young adults who follow 113 may be more consciously engage with- or may be more open about their suicidal thoughts and may handle or communicate them differently than young adults who do not follow 113 or are unaware of its existence. Furthermore, LinkedIn is primarily used for professional networking so with this recruitment strategy we might have overlooked perspectives from young adults in different socio-economic backgrounds or those less engaged in professional networking. Regarding place of residence, age, gender, education level and sexual orientation the group of participants was heterogeneously distributed. A third limitation could be the potential for response priming among participants. Since the question about needs follows questions about the general causes and factors of suicide among young adults, participants may have been influenced to respond not only based on their personal experiences but also with an awareness of suicide prevention strategies in general. Consequently, the results should be interpreted with caution, also because participants will always share their experiences and needs within the context of their own perception of the world. In the context of suicide prevention, our aim was to improve understanding the needs of young adults experiencing suicidal ideation. However, in this study, we have questioned young adults who have recently recovered from suicidal ideation regarding their fulfilled or unfulfilled needs. It is important to consider that the perspectives of young adults in partial or full recovery may differ from those actively experiencing suicidal ideation.

## 4 Conclusion

This study identified the needs and perspectives of young adults with suicidal ideation in terms of help and support to prevent or to recover from suicidal ideation. The findings underscored the importance of fostering openness and knowledge about suicide within society. Additionally, young adults reported a need for enhanced mental health education within school curricula from an early age. Furthermore, clear information and guidance on where to seek help, along with reduced barriers to accessing services, such as long waiting times, were identified as essential steps to improve mental health care seeking. Regarding informal support, participants expressed a need for easily accessible contacts in school and work environments as well as opportunities for more peer contact. Finally, addressing societal stressors, including societal welfare, academic pressure and social media, was identified as crucial for suicide prevention efforts. Structural changes should be implemented to create more supportive environments and decrease stressors contributing to mental health challenges among young adults. Overall, the results of this study highlight the importance of multi-faceted approaches to suicide prevention that address the diverse needs and perspectives of young adults with recent suicidal ideation.

## Data Availability

The raw data supporting the conclusions of this article will be made available by the authors, without undue reservation.
