# Supplementary material for: Understanding the needs and perspectives of young adults with recent suicidal ideation: insights for suicide prevention
Source: Front Child Adolesc Psychiatry. 2024 Jun 12;3:1376872. doi: 10.3389/frcha.2024.1376872 (PMC11731987; doi:10.3389/frcha.2024.1376872)
Supplement: Supplementary file 1 [file Datasheet1.pdf]

Appendix 1

Interview topic guide

1. Personal experience with suicidality
   Can you tell us about your own experience with suicidality?
   <Summarize and probe further, think about: origin, causes, course>

2. Causes and factors of suicidality in general
   According to you, what could be significant causes of the high number of suicides among young adults between the ages of 20 and 30? (especially considering the past 2 years)
   <Summarize and probe further>

3. Needs
   We'd like to discuss with you about your needs in terms of support or help. Could you tell what has helped you during the period of suicidality? Are there things you would have needed or missed during the period of your suicidal thoughts?
   <Summarize and probe further, think about; what did this look like, why did it help or not, what was the reason this wasn't present>
